# A new optical leaf-clip meter for simultaneous non-destructive assessment of leaf chlorophyll and epidermal flavonoids

**DOI:** 10.1111/j.1399-3054.2012.01639.x

**Published:** 2012-11

**Authors:** Zoran G Cerovic, Guillaume Masdoumier, NaÏma Ben Ghozlen, Gwendal Latouche

**Affiliations:** a Laboratoire Écologie Systématique et Évolution, Université Paris-SudUMR 8079, Bât. 362, Orsay, F-91405, France; b CNRSOrsay, F-91405, France; c AgroParisTechParis, F-75231, France; d FORCE-A, Université Paris-SudBât. 503, Orsay, F-91405, France

## Abstract

We have characterized a new commercial chlorophyll (Chl) and flavonoid (Flav) meter called Dualex 4 Scientific (Dx4). We compared this device to two other Chl meters, the SPAD-502 and the CCM-200. In addition, Dx4 was compared to the leaf-clip Dualex 3 that measures only epidermal Flav. Dx4 is factory-calibrated to provide a linear response to increasing leaf Chl content in units of µg cm^–2^, as opposed to both SPAD-502 and CCM-200 that have a non-linear response to leaf Chl content. Our comparative calibration by Chl extraction confirmed these responses. It seems that the linear response of Dx4 derives from the use of 710 nm as the sampling wavelength for transmittance. The major advantage of Dx4 is its simultaneous assessment of Chl and Flav on the same leaf spot. This allows the generation of the nitrogen balance index (NBI) used for crop surveys and nitrogen nutrition management. The Dx4 leaf clip, that incorporates a GPS receiver, can be useful for non-destructive estimation of leaf Chl and Flav contents for ecophysiological research and ground truthing of remote sensing of vegetation. In this work, we also propose a consensus equation for the transformation of SPAD units into leaf Chl content, for general use.

## Introduction

The Dualex 4 Scientific (Dx4) is the last generation of chlorophyll (Chl) meters that appeared recently on the market. It is a three-in-one instrument, meant to replace the Dualex 3 (FORCE-A, Orsay, France), that measures leaf epidermal flavonoids (Flav) at 375 nm, using the Chl fluorescence screening method (Bilger et al. 1997, Cerovic et al. 2002, Agati et al. 2005). In addition to Flav, Dx4 can perform measurements of Chl content from leaf transmittance, and it has an incorporated GPS for geolocalization. This device has not yet been compared to other available Chl meters, nor have its characteristics been published.

The use of leaf-clip-type sensors to assess Chl content from apparent leaf transmittance can be traced to the work of Inada (1963). It was followed by a series of commercial devices: Fuji GM-1 Chlorophyll meter and Minolta SPAD-501 that are not produced any more, SPAD-502 (SPAD hereafter) and Hydro N-tester both produced by Minolta (Konica-Minolta, Tokyo, Japan), CCM-200 (CCM hereafter) (Opti-Sciences, Hudson, NH), CL-01 (Hansatech, King's Lynn, United Kingdom) and Dualex 4 Scientific (FORCE-A, Orsay, France).

Although a substantial literature exists on the characteristics of various Chl meters, with repeated attempts to produce a reliable calibration curve for quantitative estimation of leaf Chl from sensors indices, the question of the ‘transfer’ model is still not settled. Leaf-clip Chl meters indices have no units and they are affected by leaf anatomical characteristics as shown for example by Marenco et al. (2009) for leaf thickness, fresh leaf mass per area (succulence), dry leaf mass per area (LMA) and leaf water content, and by Yamada and Fujimura (1991) for cuticle reflectance. Proportion of leaf veins (Uddling et al. 2007) and the flatness of the leaf can also influence the measurements. Most of these anatomical effects can be grouped under the concepts of sieve effect (transmittance larger than in a homogenous sample) and detour effect (absorptions larger than in a homogenous sample due to increased light-pass) (cf. Vogelmann 1993) modeled recently for SPAD by Uddling et al. (2007). So, each sensor type has to be calibrated for the plant species of interest by extractive wet chemistry (e.g. Markwell et al. 1995).

It has been shown that SPAD readings can be influenced by environmental and measurement conditions. For example, the recorded diurnal changes in SPAD reading (Hoel and Solhaug 1998) were explained by light-dependent chloroplast movements (Nauš et al. 2010). The accuracy of sensor indices is degraded by very large leaf Chl contents (Imanishi et al. 2010). Significant differences were found between two instruments both for SPAD-501 (Marquard and Tipton 1987) and SPAD-502 (Markwell et al. 1995) leading the authors to propose that each instrument should be calibrated individually against extracts. Usually non-linear relationships were found for the response of most leaf clips (cf. CCM-200 vs. SPAD) (Richardson et al. 2002). Efficiency of leaf extraction by organic solvents is also a potential problem for calibration of sensors (Lashbrooke et al. 2010).

Irrespective of all these problems, non-destructively measured leaf Chl content was used as a surrogate for leaf nitrogen (N) in plant productivity research (e.g. Pinkard et al. 2006) and as an indicator of N nutrition of crops (Cartelat et al. 2005, Yu et al. 2010, Tremblay et al. 2012). This topic is beyond the scope of this report, but it is important to mention the advantage of the concomitant measurement of both leaf Chl and Flav that is available in Dx4. For the use of Chl as a surrogate of leaf N content there is often the problem of unknown LMA. Optical methods yield an estimation of Chl content on a surface basis that is therefore dependent on the LMA when the correlation with mass based nitrogen content (%N) is being sought for (Peng et al. 1993). The Chl/Flav ratio was proposed as a solution to this particular problem because the unit of expression becomes irrelevant. This new index called NBI (nitrogen balance index) is more of an indicator of C/N allocation changes due to N-deficiency than a measure of leaf nitrogen content per se (Cartelat et al. 2005). An alternative view would be to consider Flav as a surrogate of LMA. Indeed, there is a very good correlation between Flav and LMA because both are controlled by the irradiance under which the leaf was grown (Meyer et al. 2006), so the Chl/Flav ratio would correspond to an LMA-corrected Chl, i.e. Chl on mass basis.

This paper is the first to describe a new type of leaf clip that measures simultaneously Chl and epidermal Flav. The objectives of this work are therefore threefold: (1) to evaluate the metrological performances of Dx4, both for Chl and Flav estimation, (2) to compare it to existing leaf-clip Chl meters and (3) to give insights into the origins of observed differences in calibration curves among sensors.

## Materials and methods

### Plant material

Leaves were collected from field-grown and greenhouse-grown plants in Orsay France (Long. 2.183°E, Lat. 48.700°N). Four plant species were used: two dicots, outdoor-grown kiwi [*Actinidia deliciosa* (A. Chev.) C. F. Liang & A. R. Ferguson] and grapevine (*Vitis vinifera* L.) cv. Merlot and Cabernet Sauvignon, and two monocots, wheat (*Triticum aestivum* L.) (from the greenhouse) and maize (*Zea mays* L.) (grown both in the field and the greenhouse).

### Sensor measurements and pigment analysis

Discs of 16 mm diameter (2 cm^2^) were used for the extraction. The exact point where sensor measurements were performed was sampled in order to mitigate for leaf heterogeneity. The sensing surface of SPAD is 2 mm × 3 mm, compared to a 10 mm diameter for CCM (Opti-Sciences, Hudson, NH ) and a 6 mm diameter for Dx4 (FORCE-A, Orsay, France). For chlorophyll estimation, measurements were always performed with the adaxial leaf side facing the light sources. Leaf discs were collected immediately after measurements, frozen in liquid nitrogen and stored at – 80°C until further processing. Discs were powdered in liquid nitrogen and extracted three times with methanol (3 × 1.5 ml) containing CaCO_3_. Supernatants of the three centrifugations (10 000 *g*, 5 min) were grouped and topped to 5 ml, then centrifuged again at 4100 *g* for 5 min. The extinction coefficients for pure methanol of Porra et al. (1989) were used to calculate the Chl concentration in the extracts (in µg cm^–2^):


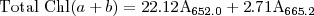
(1)

where A stands for absorbance in a 1-cm cuvette at the specified wavelength (spectrophotometer HP 8453, Agilent, les Ulis, France).

LMA was estimated for each leaf by sampling a second 16-mm-diameter disc adjacent to the one used for Chl estimation. The disc was dried at 60°C for 48 h and weighed.

### Transformation of sensor's signals into chlorophyll units

Sensor's signals are transformed to index units displayed on the leaf-clip sensor by the formula (Uddling et al. 2007, Nauš et al. 2010)


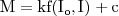
(2)

where f(I_o_,I) is the function by which the index is calculated from the signals; ‘k’ is the proportionality constant to obtain Chl in units of µg cm^–2^, and ‘c’ is a constant that corrects for the potential bias of the model. The function for Dx4 is (FORCE-A personal communication)


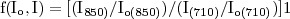
(3)

and for SPAD (Minolta 1989, Uddling et al. 2007, Nauš et al. 2010)


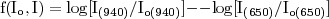
(4)

where I_o_ are signals without the leaf sample, I signals with the sample present in the leaf clip. Subscript values are wavelengths in nanometer, and log is the decadic logarithm. The transformation formula for CCM is unknown.

### Statistical analysis and data elaboration

Data were treated, transformed, statistically analyzed, fitted and plotted using a combination of software: excel 2003 (Microsoft, Redmond, Washington), statistica 6 (StatSoft, Tulsa, OK) and Igor Pro 6.02 (WaveMetrics, Lake Oswego, Oregon). The accuracy of the fitted models and of the prediction of Chl content was assessed by the coefficient of determination (R^2^), the residual sum of squares (RSS), the root mean square error (RMSE), the BIAS and by the standard error of prediction corrected for the BIAS (SEPC)


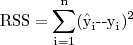
(5)


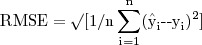
(6)


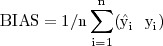
(7)


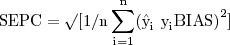
(8)

where ŷ_i_ is the model predicted value and y_i_ the measured value.

## Results and discussion

### Dualex 4 Scientific leaf-clip description and characteristics

The Dx4 leaf clip displays three values on the screen: ‘Chl’ for the Chl index, ‘Flv’ for the Flav index and ‘NBI’. The latter is the ratio of Chl and Flav (see section Introduction). ‘Flv’ and Flav stand for flavonoids because flavones (in monocots) and flavonols (in dicots) are the major epidermal phenolic absorbers at 375 nm (Cerovic et al. 2002) the wavelength used in Dx4.

The manufacturer states a repeatability of 1.3 and 2.5%, and a reproducibility of 4.5 and 3.5% (percent standard deviation) for Chl and Flav, respectively (FORCE-A 2011). Data presented in Table 1 show that in our hands both the repeatability (0.62% for Chl and 0.22% for Flav, single sensor) and the reproducibility (2.4% for Chl and 3.4% for Flav, among sensors) were better than the manufacturer's specifications. Most striking was the good repeatability for Flav measurements both for a closed clip and with reopening. The fourfold increase in variation (0.16–0.62%) for Chl measurements with reopening might be due, at least in part, to leaf heterogeneity. Judging from the inter-instrument comparison, Dx4 is rather precise both for Chl and Flav estimation (around 3% error).

**Table 1:** Major measurement characteristics of Dualex 4 Scientific. Repeatability (instrumental variations) was evaluated by the standard deviation (sd) and percent standard deviation (%sd) for 30 consecutive measurements on a leaf with mean Chl value of 21 µg cm^−2^ and mean Flav absorbance of 1.8. Reproducibility (inter-instrument agreement) was obtained from the measurements among five different Dx4 on 80 leaves of four different species. Accuracy for Chl was estimated from the calibration against Chl extracts: root mean square error (RMSE) from Fig. 3 (N = 195) and percent RMSE (%RMSE) for a mean Chl value of 32 µg cm^−2^. Accuracy for the Flav index was estimated from the comparison to Dualex 3 in absorbance units: RMSE from Fig. 2 (N = 74) and %RMSE for a mean Flav absorbance equal to 1.2

	Chlorophyll		Epidermal Flavonoids		NBI	
	
Source of variation	sd	%sd	sd	%sd	sd	%sd
Repeatability						
Clip closed	0.034	0.16	0.004	0.22	0.037	0.31
Clip opened between	0.132	0.62	0.004	0.22	0.063	0.53
	RMSE	%RMSE	RMSE	%RMSE		
	
Reproducibility	0.713	2.4	0.034	3.4		
Accuracy	5.03	16	0.185	15		

**Fig. 1:**
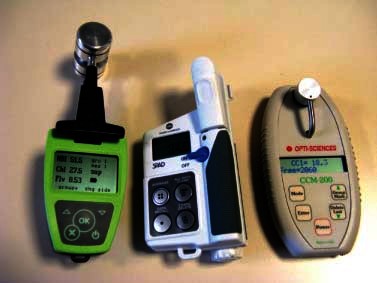
Leaf-clip sensors used in this study. From left to right: Dualex 4 Scientific, SPAD-502 and CCM-200.

**Fig. 2:**
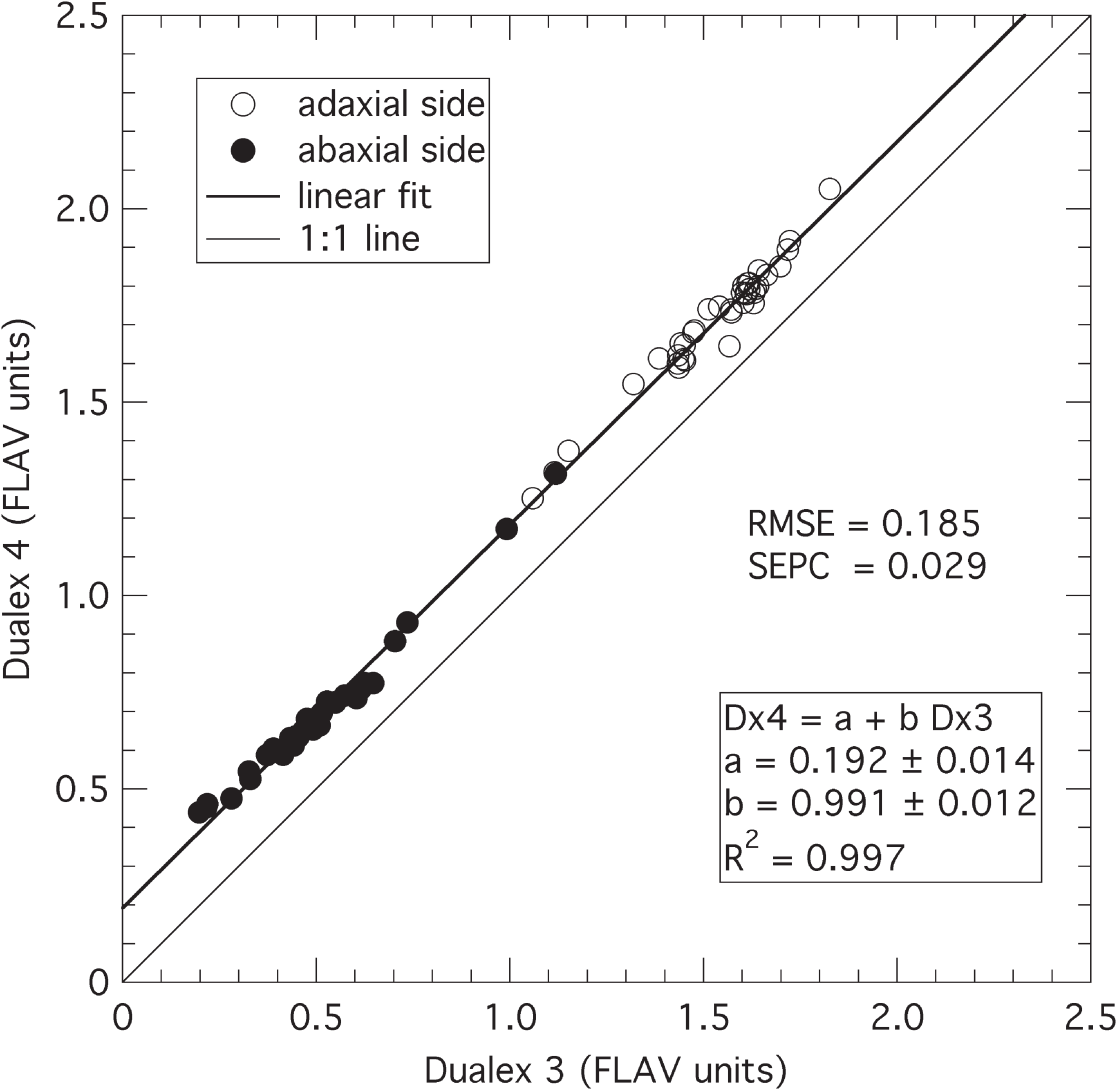
Comparison of the flavonoid meter function of Dualex 4 Scientific to the Dualex 3. Adaxial and abaxial sides of grapevine leaves were measured with the two devices, and a linear model was fitted. RMSE and SEPC are indicated in the graph along with the coefficient values of the model with their ±95% confidence intervals.

The estimation of the accuracy of the sensor, 16% for Chl and 15% for Flav, was more difficult to obtain because of the lack of a reliable reference method. Accuracy for Chl was estimated from the calibration against extracted Chl (see below). But because of the uncertainty of extraction as a reference method and the differences among plant species, we also compared Dx4 with two existing leaf-clip Chl meters (Fig. 1). For the Flav, there is no reference method due to the difficulty to isolate the epidermis. So again the estimation of accuracy relied on the comparison to the previously commercialized sensor Dualex 3. The presence of a bias when compared to a Dualex 3 (Fig. 2) explains the apparent low accuracy (15%). When this bias is taken into account the accuracy (SEPC = 2.9%) is of the same level as the reproducibility among instruments (%sd = 3.4%).

### Calibration for epidermal flavonoids

The family of Dualex leaf clips was originally designed for the measurement of epidermal Flav based on the ultraviolet (UV)-screening effect they procure to the underlying Chl in the leaf mesophyll (Goulas et al. 2004). By equalizing Chl fluorescence under visible (650 nm) and UV (375 nm) light excitation, thanks to the use of an electronic feedback loop, variable Chl fluorescence is avoided and a precise measurement of the absorbance of Flav in the UV-A is secured (Goulas et al. 2001). The Dx4 Flav values, by definition, have no unit because they represent a ratio. They could be calibrated by comparison to another accepted standard procedure for the estimation of epidermal Flav constituents. These standard techniques often involve extraction followed by spectroscopic or high performance liquid chromatography analysis of the extracts. But, the problem is how to isolate the epidermis. The comparison of Dx4 with Dualex 3 for epidermal Flav measurements is presented instead (Fig. 2). Both the regression coefficient and the slope (0.997 and 0.991, respectively) demonstrated a very reliable correspondence between the two sensors. Still, a significant bias of 0.19 absorbance units of Flav was present. It can be traced to the difference in factory calibration (FORCE-A personal communication) and to the choice of the reference standard without epidermal absorbance. Ideally it should be a leaf without epidermis with an equal excitation of Chl fluorescence at 650 nm (red) and 375 nm (UV-A) (Barnes et al. 2000). Markstädter et al. (2001) propose to divide the measurements on the sample by the identical measurement (same device, same conditions) of peeled leaves or, if leaf peeling is not possible, a blue foil standard that has fluorescence properties similar to that of leaves (Kolb et al. 2001, Markstädter et al. 2001). The standard blue fluorescent foil used by FORCE-A has different spectral characteristics than Chl in vivo. The factory correction for this discrepancy is therefore crucial for instrument agreement and accuracy of the Flav estimation. We tend to favor the Dx4 factory calibration over that of Dualex 3 because it showed a zero Flav value on leaves with the epidermis stripped off (data not shown). On the same samples Dualex 3 showed negative values.

Apart from the described zero shift, the correspondence between Dualex 3 and Dx4 for epidermal Flav measurements was very good even though Dualex 3 is an all-analogical instrument compared with the all-digital Dx4 instrument. Still, the critical parts have not been changed: the LED light sources defining the sampling wavelengths remained at 375 nm in the UV and 650 nm in the red.

### Calibration for chlorophyll and comparison to two other sensors

We then characterized the accuracy of the Chl meter function of Dx4. The manufacturer of Dx4 claims a linear response of the sensor to leaf Chl content and a direct equivalence between Dx4 units and units of total Chl in µg cm^–2^. To verify this, we made a calibration against a standard procedure for Chl extraction, concomitant to a comparison of Dx4 to two other Chl meters: the SPAD and the CCM. Common leaf discs were used to calibrate all three sensors against extracted Chl.

**Fig. 3:**
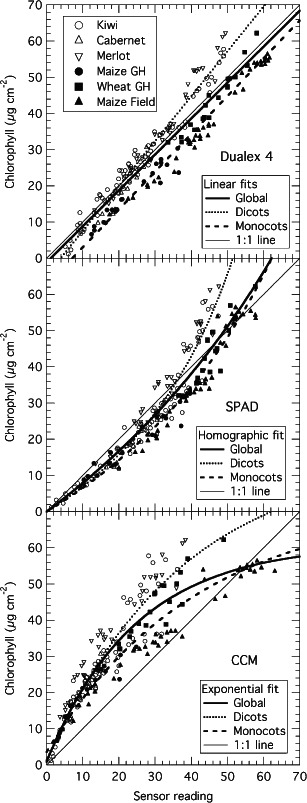
Calibration of the three sensors against the chlorophyll extracts. Dicot plants are indicated with open symbols and monocots with closed symbols. All dicot leaves came from the field and monocot leaves were either from greenhouse (GH) grown plants or from the field. Fits for global models encompassing all data points are plotted along with the fits for the dicot and monocot plants independently: for Dualex linear (a + bx), for SPAD homographic ((ax)/(b – x)) and for CCM exponential (a + be^cx^); *P* < 0.0001 for all models. Coefficients of the models are presented in Table 2.

Numerical values for the Dx4 and SPAD readings were close to the leaf Chl content on a surface basis in units of µg cm^–2^ (Fig. 3). Sensor readings for CCM were much smaller. Fig. 3 and Table 2 show that the Dx4 response to leaf Chl was linear. The response of SPAD readings was slightly concave and that of CCM readings largely convex as often reported in the literature (Markwell et al. 1995, Castelli et al. 1996, Richardson et al. 2002, Cartelat et al. 2005, Uddling et al. 2007, Coste et al. 2010). The SPAD and CCM need, therefore, to be fitted with non-linear functions (Table 2). Fitting of several functions (see Table S1) and using the principle of parsimony (favoring models with the least number of parameters) (Legendre and Legendre 1998) showed that the more appropriate models were linear for Dx4, homographic (Coste et al. 2010) for SPAD and exponential for CCM (Fig. 3). The advantage of a linear sensor response over a curvilinear response was already discussed by Richardson et al. (2002). The same authors have found a smaller relative error of Chl prediction for SPAD (19%) than for CCM (20%). We found a relative error of 16% for Dx4, 16% for SPAD and 27% for CCM, which confirms the finding of Richardson et al. (2002) and shows that Dx4 has the same level of accuracy as SPAD.

**Table 2:** Characteristics of the calibration models for the three sensors. Models were parameterised for all data points or by separating the dicot (grapevine and kiwi) and monocot (wheat and maize) species (*P* < 0.0001 for all models). They were linear (a + bx) for Dualex, homographic [(ax)/ (b − x)] for SPAD and exponential (a + be^cx^) for CCM. The 95% confidence intervals for the fit coefficients are indicated in brackets (non-significant in italic). Residual sum of squares (RSS), root mean square error (RMSE), bias (BIAS), standard error of prediction corrected for bias (SEPC), relative error (%) = SEPC/mean are given

Sensor Species	Model parameters			Model statistics					Mean	Min	Max		
			
	a	b	c	R^2^	RSS	RMSE	BIAS	SEPC	(µg cm^−2^)			N	Error (%)
**Dualex** Monocots	−7.46 (2.0)	1.04 (0.046)	−	0.963	631	6.36	−5.67	2.89	39.9	12.4	61.6	79	7
Dicots	−4.82 (1.4)	1.24 (0.047)	−	0.960	960	4.24	1.57	3.94	26.7	5.18	49.5	117	15
Global	−*1.12 (1.8)*	0.993 (0.051)	−	0.883	4952	5.20	−1.34	5.03	32.0	5.18	61.6	196	16
**SPAD** Monocots	82.2 (10)	135 (11)	−	0.941	1000	3.56	−0.06	3.56	38.2	9.40	57.8	79	9
Dicots	59.0 (6.1)	95.0 (5.8)	−	0.915	2026	4.16	−0.12	4.16	29.1	1.30	47.6	117	14
Global	138 (47)	185 (48)	−	0.876	5269	5.18	0	5.18	32.7	1.30	57.8	196	16
**CCM** Monocots	72.4 (6.8)	−68.8 (5.8)	−0.0242 (0.0045)	0.913	1484	4.33	0	4.33	27.5	2.90	63.1	79	16
Dicots	86.1 (14)	−84.9 (13)	−0.0267 (0.0070)	0.897	2440	4.57	0	4.57	15.2	1.20	38.4	117	30
Global	61.1 (5.7)	−60.2 (4.6)	−0.0407 (0.0089)	0.863	5804	5.44	0	5.44	20.1	1.20	63.1	196	27

The origin of these differences in sensor responses can be due to factory calibration procedure performed by the manufacturer to transform sensor signals into indices, or it could stem from the use of a different form of indices (compare Eqn 3 with Eqn 4) and sampling wavelengths used in Dx4. Even for the same index, the precise center wavelength and the bandwidth of the source can influence the sensor response. Moreover, Carter and Spiering (2002) found earlier that the ratio of transmittance at 710 and 850 nm (used in Dx4) correlates best with leaf Chl content in wild grape. Actually, Yamada and Fujimura (1991) had already noticed that the choice of a wavelength with large absorption coefficient for Chl (like 652 nm used in SPAD and CCM) increases the accuracy at low leaf Chl contents, but loses accuracy at high leaf Chl contents. The absorption of a typical grapevine leaf is 87% at 652 nm compared with 40% at 710 nm (Cerovic unpublished). Selecting a wavelength with a small absorption coefficient induces a lower accuracy over the whole range of Chl content, but gives indices less prone to saturation at high Chl values. The additional advantage of the 710-nm sampling wavelength is a smaller ‘sieve effect' (Vogelmann 1993) and the avoidance of interference by the presence of anthocyanins, for example, in red young leaves or temperature stressed leaves (Liakopoulos et al. 2006).

### Sources of uncertainty for chlorophyll estimation using leaf-clip sensors

The difference in sensor response for monocots vs dicots was obvious for all three sensors (Fig. 3). Table 2 shows that the prediction error is substantially decreased when the two types of plants are treated independently (Dx4 SEPC of 5.03, 3.94 and 2.89 µg cm^–2^ Chl, for the global model, the dicots and the monocots, respectively). This difference can be linked to the differences in leaf anatomy: the proportion of vascular tissue per unit surface is larger in monocots than in dicots and the latter are characterized by the presence of palisade and spongy tissue and thicker adaxial cuticle (Esau 1953, Yamada and Fujimura 1991, Cerovic et al. 1999). For a given leaf Chl content (Fig. 3, y axis) the sensor index (Fig. 3, x axis) would decrease due to the presence of sieve effect and increase due to the presence of the detour effect at the sampling wavelength in the visible, but the detour effect would produce the opposite effect at the reference wavelength, i.e. a decrease in the sensor index. Indeed, the vascular tissues increase the transmittance in monocots, especially at the reference wavelength 850 nm (not shown, see also Woolley 1971) leading to a larger sensor index. A more reflecting cuticle (Yamada and Fujimura 1991) and a larger detour effect in dicots, often linked to thicker leaves (larger LMA), increase the reflectance and decrease the transmittance, again more at the reference wavelength (not shown), which leads to a smaller sensor index. The use of a multiple regression model that included the LMA increased the quality of Chl prediction: for Dx4, the global R^2^ increased from 0.883 to 0.935 and the SEPC decreases from 5.03 to 3.78 (not shown) (for LMA data see Fig. S1). It seems that it would be advantageous to have two independent calibration curves, one for monocots and another for dicots in Dx4 and also for the two other sensors. As seen in Table 2, this would decrease the coefficient of variation from 16% for the general model to 15% for dicots and 7% for monocots.

**Fig. 4:**
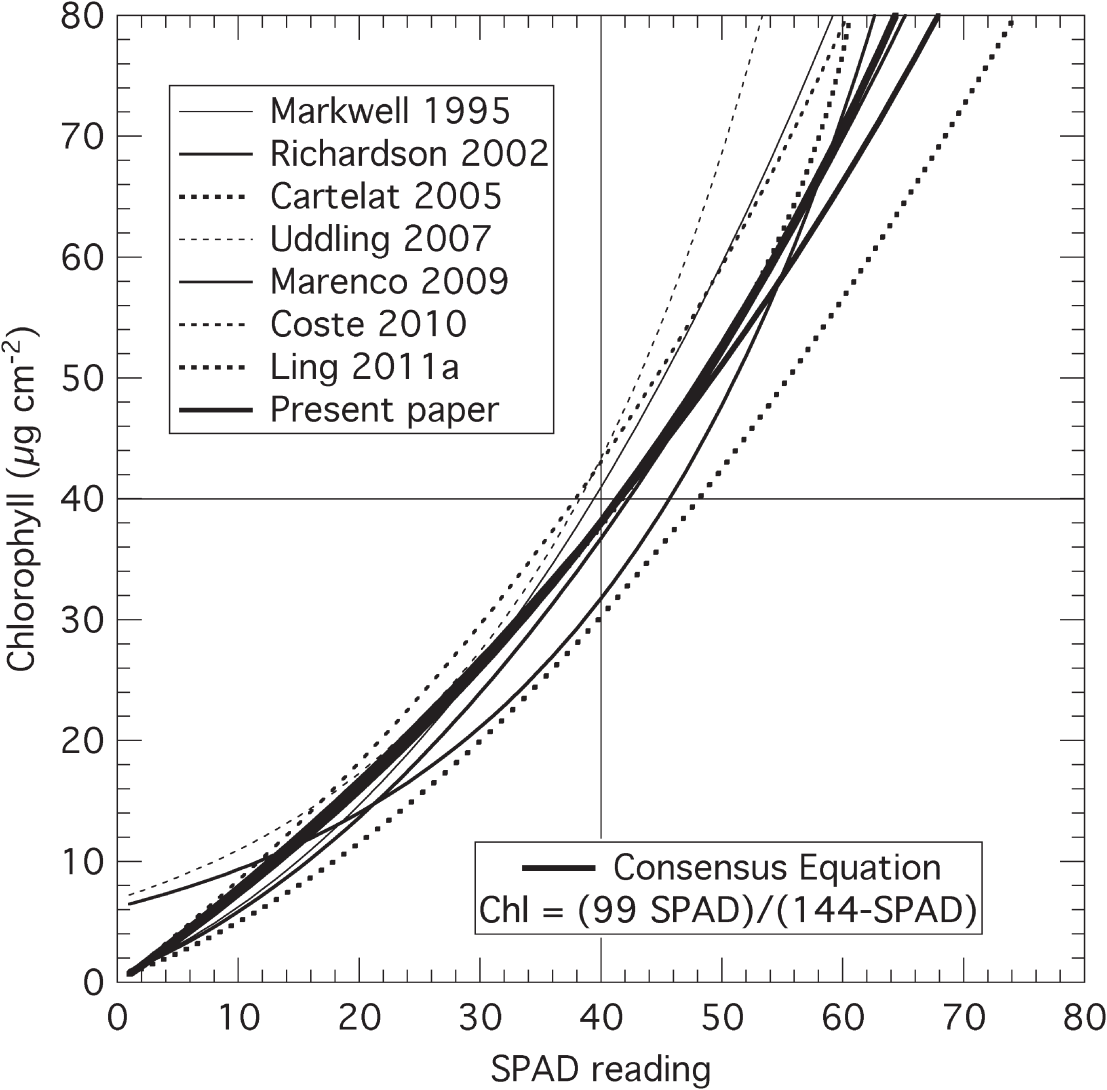
Comparison of the calibration models for SPAD-502 available in the literature. All data were adjusted to common units for Chl in µg cm^–2^. The functions for the eight models plotted on the graph from which the consensus equation was derived were: y = 0.09 10^(x∧0.265)^(Markwell et al. 1995); y = 0.552 + 0.404x + 0.0125 x^2^ (Richardson et al. 2002); y = 93.6 – 11.9 √ (62 – x) (Cartelat et al. 2005); y = 6.91 e^(0.0459x)^ (Uddling et al. 2007); y = 6.205 e^(0.0408x)^ (Marenco et al. 2009); y = (117.1x)/(148.84 – x) (Coste et al. 2010) y = 0.9 (0.381 + 0.4119x + 0.0105x^2^) (Ling et al. 2011a, 2011b); y = (138x)/(185 – x) (present paper).

The dispersion of points is much smaller when sensors are plotted against each other (Fig. S2), showing that the extraction procedure is responsible for part of the dispersion of the calibration points. The extraction method, the organic solvents used and their purity, the choice of absorption coefficients (source of reference equation) and even the characteristics of the spectrophotometer used (bandwidth, stray light) can all influence the numerical value of Chl in extracts used to calibrate the sensors (Wellburn 1994). For example, the difference in the calibration equation obtained recently by Uddling et al. (2007) using a Shimadzu spectrophotometer with a 1-nm spectral resolution and the extinction coefficients of Porra et al. (1989) compared with the one obtained by Marenco et al. (2009) using a 5-nm spectral resolution and the extinction coefficients of Arnon (1949) is already 20%, even without the influence of the extraction procedure. The extraction procedure is actually a major source of variability because it depends on the operator's skill. For all these reasons the accuracy of the numerical value among different published sources cannot be better than 10%, and is often much worse (Fig. 4).

The SPAD dominates by far the literature on the use of Chl leaf clips with 624 occurrences of the key words ‘SPAD and chlorophyll’ compared with 91 for ‘CCM and chlorophyll’ (source: web of knowledge, http://www.wokinfo.com, assessed on November 3, 2011). We therefore examined the publications reporting calibration curves for the SPAD (Fig. 4). The reported calibration models were all concave curvilinear, for a variety of plant species, sometimes with a small offset at the origin (for zero SPAD reading) (Uddling et al. 2007, Marenco et al. 2009). Data were modeled by polynomial (Richardson et al. 2002, Ling et al. 2011a, 2011b), exponential (Markwell et al. 1995, Uddling et al. 2007, Marenco et al. 2009) or homographic (Coste et al. 2010) functions. Linear functions were also proposed, but mostly when the dispersion of experimental points was too large (not shown), and these were not retained here. From the chosen eight sources (seven cited, plus our own), we drew a consensus equation for the transformation of SPAD readings into surface-based specific units of Chl (µg cm^–2^):


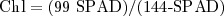
(9)

that can be very useful for the future use of SPAD.

### The usefulness of leaf-clip sensors and potential domains of application

The usefulness of non-destructive measurement of Chl is obvious. The use of optical Chl meters, whether Dx4 directly or SPAD by using the consensus equation, is probably more accurate and certainly more rapid than to rely on extractions because of the very good reproducibility of the sensors. This is especially true for mature leaves of crop plants most important for agriculture, perhaps less true for exotic species with high Chl contents and thick leaves. Nevertheless, the present absolute accuracy for Dx4 and SPAD is similar or even better than the natural variability of mature leaves. The natural variability for a mature dicot leaf, for example, for grapevine or kiwi leaves used in this study, is around ±10%. It is far larger along monocot leaves, being up to ±40% for a mature wheat or maize leaf (data not shown). So, leaf-clip Chl meters are valuable tools for the estimation of Chl thanks to their capacity for many measurements allowing one to avoid or assess the natural heterogeneity of leaves. In addition to their application in agriculture, the availability of a new calibrated sensor with a linear response to Chl will help generalize their use in ecophysiology and plant protection (e.g. Hawkins 2009). Indeed, the knowledge of the leaf Chl content is crucial in many domains of plant research and agronomical and horticultural application. Chlorophyll is an important parameter for cultivar selection and phenotyping. It is the basis for the expression of the rate of photosynthesis and, as a corollary, plant productivity. For large-scale canopy productivity estimation, often assessed through remote sensing, the use of Chl meters is crucial for ground truthing. These hand-held sensors will have the same role for the calibration of vehicle-mounted proximal sensors (Tremblay et al. 2012).

Chlorophyll is used as a surrogate for leaf nitrogen content not only in productivity studies, but even more so in agriculture as an indicator of N-deficiency. Specific sensors (e.g. the N-tester from Yara) are used as a decision support tool for N-fertilization of crops (Tremblay et al. 2012). The combination of Chl and Flav in the Dx4 now allows a more widespread use of the NBI index that has been shown superior to Chl alone for N-fertilization in several crops (Tremblay et al. 2009, Tremblay et al. 2010, 2012). Calibration of Dx4 NBI for nitrogen management of grapevine and wheat is in progress in our laboratory.

In conclusion, we have shown that the new Dualex 4 Scientific leaf-clip Chl and Flav meter is as precise and accurate as SPAD for Chl and Dualex 3 for Flav. It has a linear response to leaf Chl content and delivers readings in units of µg cm^–2^. It is a useful addition to presently available devices for non-destructive assessment of plant status, especially in regards to the simultaneous assessment of Chl and Flav on the same leaf spot.

## Abbreviations

Chl: chlorophyll
Dx4: Dualex 4 Scientific
Flav: flavonoid
LMA: dry leaf mass per area
N: nitrogen
NBI: nitrogen balance index
RMSE: root mean square error
RSS: residual sum of squares
SD: standard deviation
SEPC: standard error of prediction corrected for the BIAS
UV: ultraviolet

## Supporting Information

**Table S1.** Comparison of different model equations for the three sensors.

**Fig. S1.** Leaf mass per area (LMA) of data points used for calibration plotted against the leaf chlorophyll content.

**Fig. S2.** Direct sensor comparisons one-to-one.
