# Supplementary material for: A new optical leaf-clip meter for simultaneous non-destructive assessment of leaf chlorophyll and epidermal flavonoids
Source: Physiol Plant. 2012 Nov;146(3):251–60. doi: 10.1111/j.1399-3054.2012.01639.x (PMC3666089; doi:10.1111/j.1399-3054.2012.01639.x)
Supplement: Supplementary file 1 [file ppl0146-0251-SD1.doc]

Physiologia Plantarum - Technical Focus

**Title:**

A new optical leaf-clip meter for simultaneous non-destructive assessment of leaf chlorophyll and epidermal flavonoids

Zoran G. Cerovic^1^, Guillaume Masdoumier^2^, Naïma Ben Ghozlen^1^ & Gwendal Latouche^1^

*^1^Univ. Paris-Sud, Laboratoire Écologie Systématique et Évolution, UMR 8079, Bât. 362, Orsay, F-91405; CNRS, Orsay, F-91405; AgroParisTech, Paris, F-75231, France*

*^2^FORCE-A. Univ. Paris-Sud, Bât. 503, Orsay, F-91405*

**Supporting Information**

**Table S1.** Comparison of different model equations for the three sensors.


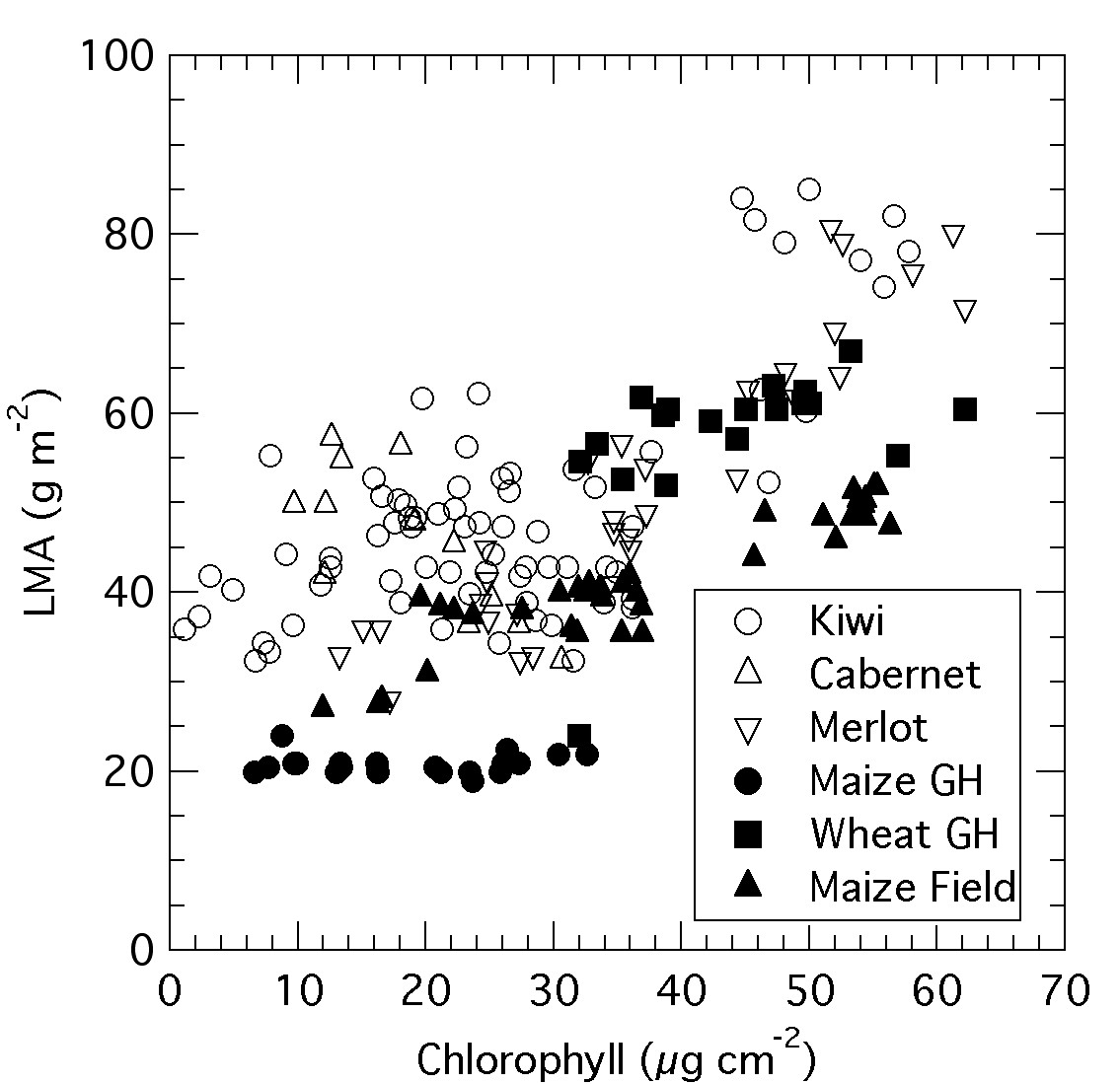


**Figure S1**. Leaf mass per area (LMA) of data points used for calibration plotted against the leaf chlorophyll content.


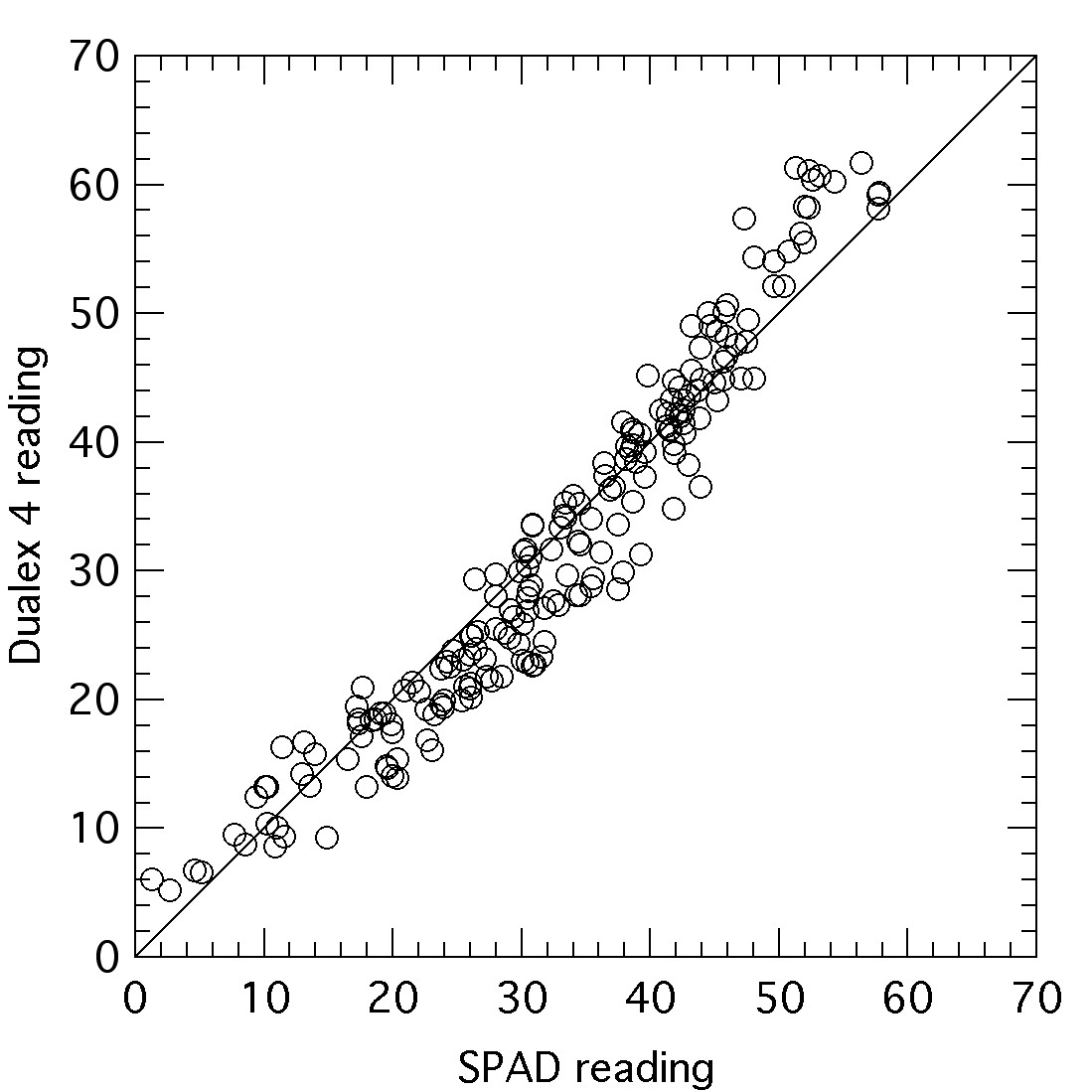

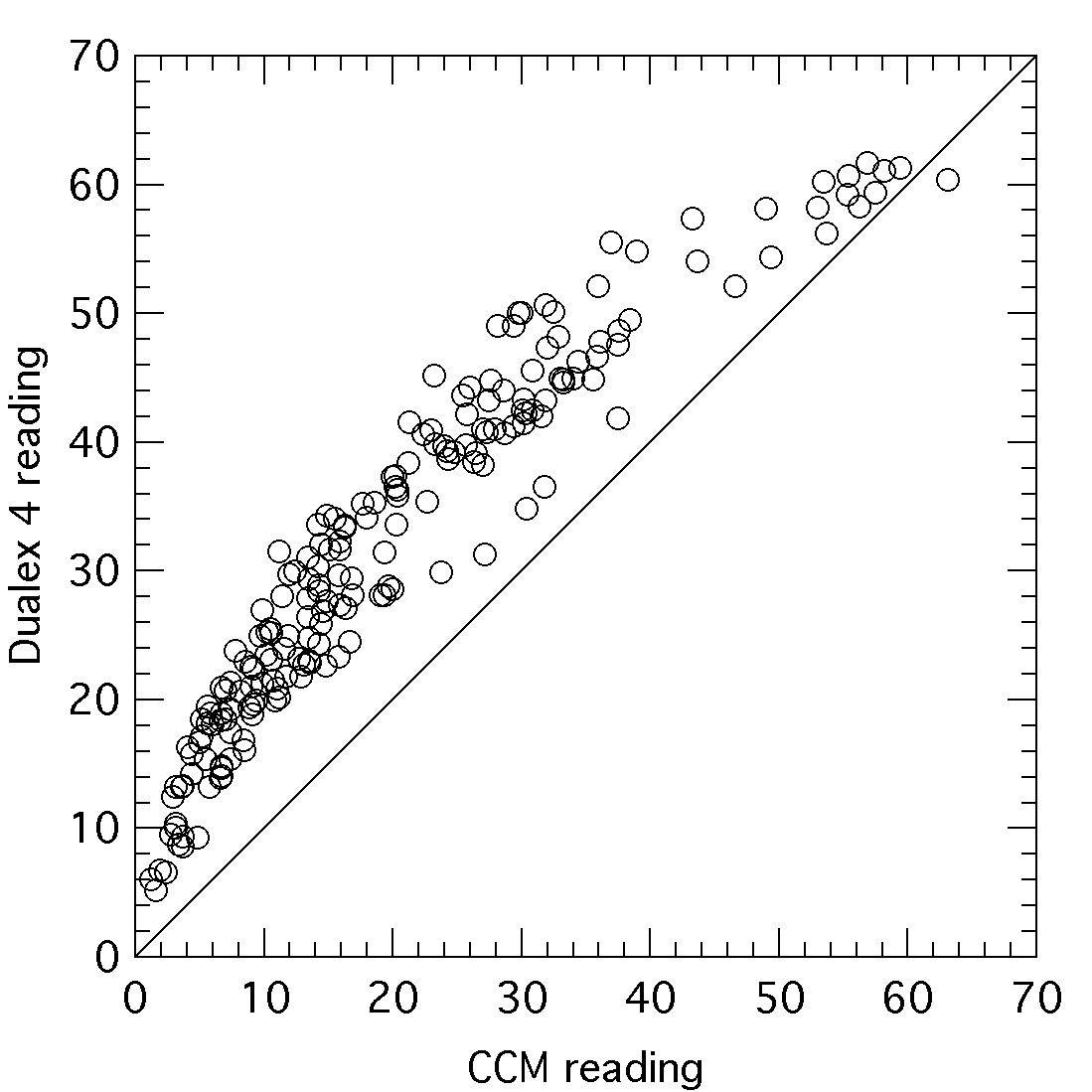

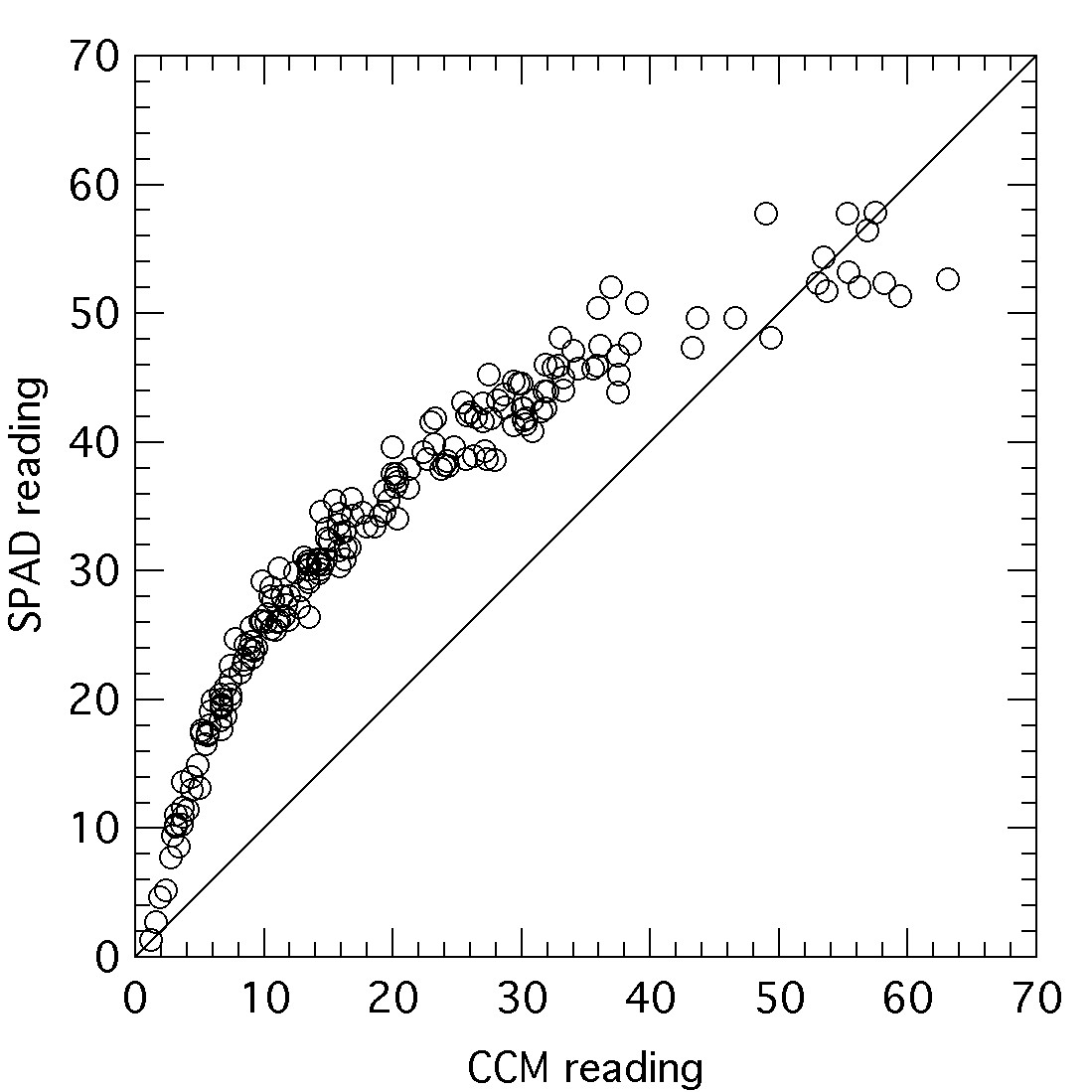


**Figure S2.** Direct sensor comparisons one-to-one.
